# Subcellular Localization of SUN2 Is Regulated by Lamin A and Rab5

**DOI:** 10.1371/journal.pone.0020507

**Published:** 2011-05-31

**Authors:** Ying Liang, Peng Hang Chiu, Kit Yan Yip, Siu Yuen Chan

**Affiliations:** Department of Paediatrics and Adolescent Medicine, Li Ka Shing Faculty of Medicine, University of Hong Kong, Pokfulam, Hong Kong SAR, China; University of Birmingham, United Kingdom

## Abstract

SUN2 is an inner nuclear membrane protein with a conserved Sad1/UNC-84 homology SUN-domain at the C-terminus. Intriguingly, SUN2 has also been reported to interact with Rab5, which localizes in early endosomes. To clarify the dual subcellular localization of SUN2, we investigated its localization in lamin A/C deficient cells rescued with lamin A or lamin C isoform, and in HeLa cells transfected with Rab5 or its mutants. We found that expression of lamin A but not lamin C partly restored the nuclear envelope localization of SUN2. SUN2 was redistributed to endosomes upon overexpression of Rab5, but remained on the nuclear envelope when the SUN domain was deleted. To explore the physiological function of SUN2 in vesicle trafficking and endocytosis, we demonstrated the colocalization of endogenous SUN2 and Rab5. Moreover, overexpression of SUN2 stimulated the uptake of transferrin while suppression of SUN2 expression attenuated the process. These findings support a role of SUN2 in endocytosis.

## Introduction

SUN2 belongs to a family of proteins with a conserved C-terminal Sad1/UNC-84 homology (SUN) domain. UNC-84 is a *Caenorhabditis elegans* protein which is required for nuclear migration and anchoring during development [1]. The nuclear envelope (NE) localization of UNC-84 depends on Ce-lamin [2]. In mammals, the SUN domain is present in six reported proteins termed SUN1, SUN2, SUN3, SPAG4/SUN4, SPAG4L/SUN5 and SPAG4L2. Proteomic screening analyses have revealed that both SUN1 and SUN2 reside on the NE [3], [4]. HA-tagged SUN3 is also localized to the NE [5], SPAG4/SUN4 is found in outer dense fiber of sperm tail [6], SPAG4L and SPAG4L2 are NE proteins specific to spermatids [7].

UNC-84, SUN1 and SUN2 have been characterized as type-II inner nuclear membrane (INM) proteins, with the C-terminal SUN domain in the perinuclear space [8], [9], [10], [11], [12]. The luminal region of UNC-84 and SUN1 serves to tether ANC-1, UNC-83 and the mammalian ANC-1 orthologs nesprins to the outer nuclear membrane through the KASH (Klarsicht, ANC-1 and Syne homology) domain [8], [9], [10], [12]. The interaction between UNC-84 and UNC-83, which is in turn connected to the cytoskeleton, is required for proper nuclear migration during development [8]. Similarly, studies on SUN1 have suggested that its luminal domain is involved in the interaction with nesprins which are connected to actin [9], [10], [12]. Recent genetic studies have revealed the roles of SUN1 and SUN2 in mice. Unlike SUN1, SUN2 loss-of-function alone does not give rise to detectable phenotype. SUN1/2 and nesprin 1/2 complexes connect centrosome to the nucleus during neurogenesis and neuronal migration [13]. SUN1 and SUN2 also play partially redundant roles in anchoring nuclei in skeletal muscle [14]. Surprisingly, SUN1 is also required for telomere attachment to nuclear envelope and gametogenesis [15]. Taken together, SUN 1 plays a more prominent role than SUN2 in nuclear architecture and positioning.

Intriguingly, SUN2 has been identified as an interacting partner of Rab5 by yeast two-hybrid screening. Truncated SUN2, therein termed rab5ip, colocalizes with exogenous Rab5 which is a small GTPase responsible for endosomal membrane fusion [16]. Interaction between SUN2 and Rab5c has also been identified in a proteomic screening [17]. There is no subsequent report in this area of SUN2 function. In the present study, we further examined the connection between SUN2 and lamins and the involvement of SUN2 in endocytosis. Our results confirm that the subcellular localization of SUN2 is regulated by lamin A and Rab5. SUN2 was redistributed to endosomes upon Rab5 overexpression and the SUN domain was essential for this process. Furthermore, colocalization between endogenous SUN2 and Rab5 could be detected. By taking the knockdown approach, our data suggest that SUN2 indeed plays a role in transferrin-receptor mediated endocytosis.

## Materials and Methods

### Plasmids

A cDNA clone containing full length human SUN2 was obtained by library screening using a partial fragment isolated previously [18]. The entire coding region of SUN2 was PCR-amplified by Elongase Enzyme (Life Technologies) using specific primers. The forward primer, 5′-ACTAAGCTTACCATGGGTTATCCATATGATGTTCCAGATTATGCTGAATTCATGTCCCGAAGAAGCCAG-3′, contained *Hind*III cutting site, Kozak sequence, start codon and hemagglutinin A (HA) epitope. The reverse primer, 5′-ACTGATATCGCAGATCCTCTTCTGAGATGAGTTTTTGTTCTGTCGACGTGTGGGCGGGCTCCCCATG-3′, included *EcoR*V cutting site and myc epitope. The purified PCR product was cloned into pcDNA3 (Invitrogen) by *Hind*III/*EcoR*V sites to give pcDNA3-HA-SUN2-myc and confirmed by sequencing. The SUN domain deletion construct pcDNA3-HA-ΔSUN was generated by digestion of pcDNA3-HA-SUN2-myc with *Sac*II and *EcoR*V and re-ligation. The luminal region deletion construct pcDNA3-HA-SUN2ΔL was generated by partial digestion with *Sma*I and ligation to the downstream *EcoR*V site.

Plasmids of pEGFP-Rab5 and its mutants were kindly provided by Dr. Brian J Knoll (University of Houston, USA). For FRET assay, the insert of pEGFP-C1-Rab5^Q79L^ was released and subcloned into pECFP-C1 (Clontech) to give pECFP- C1-Rab5^Q79L^. pECFP-C1-Rab5 was a gift from Prof. Marino Zerial (Max Planck Institute of Molecular Cell Biology and Genetics, Germany). Full length SUN2 was cloned into pEYFP-C1 (Clontech) to give pEYFP-SUN2. All plasmids were verified by sequencing.

To suppress endogenous SUN2 expression, a pair of 64 oligonucleotides containing sequences specific to SUN2 mRNA, 5′-GACTCAGAAGACCTCTTCA, was designed by Basic siRNA Design Tool (www.qiagen.com). The sequence has no similarity to SUN1. After annealing, oligonucleotides were inserted into pSUPER-EGFP, a gift from Dr. Chi-Chung Hui (The University of Toronto, Canada). The pSUPER-EGFP vector was modified from pEGFP-C1 (Clontech) by subcloning a polymerase III H1-RNA gene promoter from pSUPER vector as previously reported [19]. The accuracy of the insert was confirmed by sequencing.

### Antibodies

Rabbit polyclonal antibody to human SUN2 was generated against a synthetic polypeptide (Invitrogen) corresponding to amino acids 295–313 of SUN2 (NP_056189) according to a previous study [16]. The antiserum was affinity purified using AminoLink Immobilization Kit (Pierce). Polyclonal antibodies against HA, Rab5 and EEA1 were purchased from Santa Cruz Biotechnology (Santa Cruz). The anti-GFP antibody was supplied by Abcam (Cambridge), anti-myc antibody from Roche (Indianapolis). For colocalization of endogenous proteins, monoclonal anti-Rab5 (Sigma) was used.

### Cell culture, transient transfection and immunocytochemistry

HeLa cells were maintained in DMEM (Gibco BRL) supplemented with 10% FCS (Wisent). *Lmna ^+/+^*, *Lmna ^−/−^* embryonic fibroblasts and plasmids pEGFP-Lamin A or pDs-Red-Lamin C were obtained from Dr. Zhongjun Zhou (The University of Hong Kong, Hong Kong). Cells were cultured in DMEM containing 10% FCS and early passages (p1–p6) were used.

For transient transfection, cells were plated onto coverslips coated with 0.1% gelatin and transfected using lipofectamine (Invitrogen). After 48 hours, cells were fixed in 4% PFA/PBS followed by immunostaining. After fixation, cells were probed with antibodies and examined under Axioplan-2 epifluorescence microscope (Zeiss) using filters with excitation/emission wavelength at 480/535 nm and 540/605 nm or LSM510 Meta laser scanning confocal microscope (Zeiss) with laser wavelength: argon 488 nm and HeNe 633 nm. The setting of the confocal microscope is 63X or 100X oil objective lens; numerical aperture 1.4; pinhole size 94 µm for green and 131.1μm for red; Gain 94 %; offset from −5 to −8; pixel dimension 1024X1024; filter setting BP500–530 for green and BP650–710 for red.

### Fluorescence Resonance Energy Transfer (FRET)

HeLa cells were transfected with pEYFP-SUN2 together with pECFP-C1-Rab5 or pECFP-C1-Rab5^Q79L^. After 24 hours of transfection, cells were fixed with 4% PFA/PBS for 20 minutes. ECFP, EYFP and FRET signals were detected by LSM510 Meta laser scanning confocal microscope (Zeiss) with 63x oil-immersion lens. ECFP channel was excited by argon laser line at 458 nm and detected with band pass filter at 462–505 nm. EYFP channel was excited by argon laser line at 514 nm and detected with band pass filter at 516–548 nm. FRET channel was excited by argon laser line at 458 nm and detected with band pass filter at 516–548 nm.

### Protein extraction, immunoprecipitation and immunoblotting

To extract total protein, cells were lysed in lysis buffer (10 mM Tris pH 8.0, 1 mM EDTA, 1 mM EGTA, 0.5% Nonidet P-40, 1% Triton X-100, 150 mM NaCl, 100 µg/ml PMSF, 100 µg/ml Na_3_VO_4_ and 1 µg/ml aprotinin). Total protein (500 µg) was mixed with 1 µg of precipitating antibody in lysis buffer and then incubated at 4°C for 4 hours with gentle rotation. Protein G-plus agarose beads (Santa Cruz) or Dynabeads M-280 sheep anti-rabbit IgG (Invitrogen) were added to the mixture and then further incubated overnight. The mixture was washed with lysis buffer at 4°C for 6 times, or 3 times if Dynabeads were used.

Subcellular fractionation experiment using sucrose float-up gradients was performed according to Harley et al. 2001 [20]. Immunoblotting was performed according to standard procedures. Samples were separated by 7.5% or 12% SDS-polyacrylamide gel electrophoresis, transferred to a Protran nitrocellulose membrane (Schleicher and Schuell), and detected by enhanced chemiluminescent reagent (Amersham Pharmacia Biotech).

### Transferrin uptake assay

Transferrin uptake assay was performed as previously reported [21]. In brief, HeLa cells were plated and transiently transfected. After 48 hours, cells were washed with DMEM twice and serum starved for 3 hours. Texas Red or Alexa 632 labeled-Tf (Molecular Probe) was added to the cells at 50 µg/ml in DMEM. Cells were maintained in the dark at 4°C for 1 hour and then incubated at 37°C for 30 minutes. Uptake of Tf was stopped by washing with ice-cold PBS for 6 times. After fixation, cells were counterstained with DAPI and mounted on slides. Samples were examined under an Axioplan-2 epifluorescence microscope or LSM510 Meta NLO multiphoton confocal laser microscope (Zeiss). On each slide, three randomly selected fields with a total of 15–20 cells were analyzed. Fluorescence quantifications were determined by the software LSM510-Expert (Zeiss) on confocal images. The EGFP-fluorescence was used to identify transfected cells and the Alexa 632-intensity in the marked area of each cell was measured. The total number of slides counted for three independent transfections with pEGFP, pEGFP-SUN2 and pSUPER-EGFP-SUN2 were 6, 3, and 8 respectively. One way ANOVA with post Tukey test was used for statistical analysis.

## Results

### Lamin A but not lamin C contributes to the NE localization of SUN2

**Figure 1:**
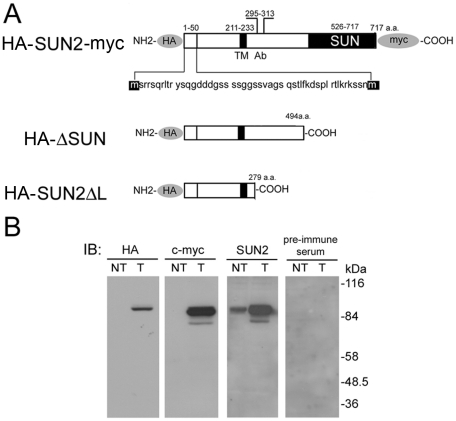
Expression constructs and antibodies for SUN2. (A) Schematic diagram of recombinant SUN2. Full length SUN2 contains a transmembrane domain (TM) and a conserved SUN domain. Each domain is labeled according to human UNC-84 homolog B (NP_056189) numbering. The N-terminal 1–50 amino acid sequence of SUN2 is also shown. The two methionine residues corresponding to the two potential translation initiation sites are highlighted in black. The position of the peptide used to generate the anti-SUN2 antibody is indicated as Ab. Regions shown in light gray are the locations of two epitopes, hemagglutinin A (HA) and myc. The construct lacking the SUN domain is labeled as ΔSUN. The construct lacking most of the lumen region C-terminal to TM is labeled as SUN2ΔL. (B) Total protein extracts (50 µg) from non-transfected HeLa cells (NT) or cells transfected with pcDNA3-HA-SUN2-myc (T) were analyzed by antibodies against HA, c-myc, SUN2 and its pre-immune serum. A dominant band at ∼85 kDa is found in all T lanes except immunoblotting with the pre-immune serum. Antibodies against c-myc and SUN2 also recognize an additional minor band at 80 kDa. Endogenous SUN2 as shown in the SUN2-NT lane is ∼85 kDa. IB, immunoblotting.

To detect endogenous SUN2 expression, we raised an antiserum against SUN2 and examined its specificity after affinity purification. HeLa cells were transfected with a plasmid expressing HA and myc-tagged full length SUN2 (Fig. 1A). Lysates of both transfected and nontransfected cells were analyzed with antibodies against HA, c-myc and SUN2. As shown in Figure 1B, all the three antibodies, but not the pre-immune serum, recognized the same band at ∼85 kDa. This indicates that SUN2 antibody specifically recognized SUN2. In the transfected cells, a less prominent band at 80 kDa was also detected with antibodies against c-myc and SUN2. Possible explanations include alternative splicing, alternative initiation at the second methionine codon (in the +50 position) (Fig. 1A) or incomplete posttranslational modifications.

**Figure 2:**
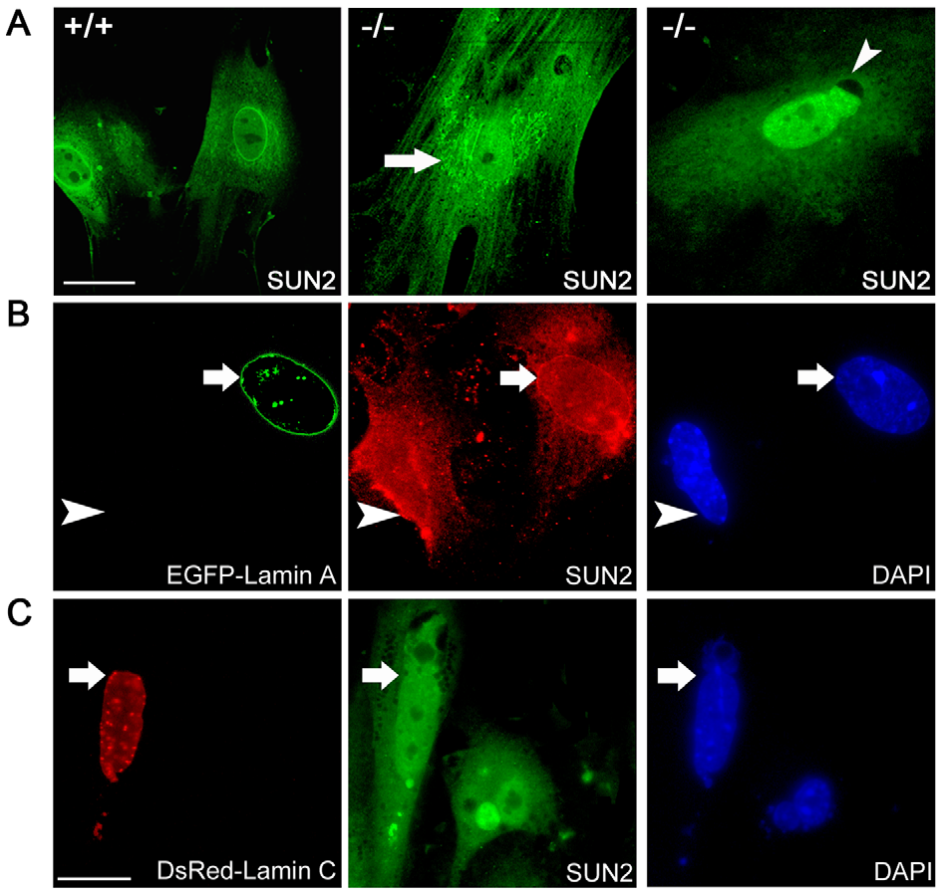
SUN2 retention on the NE is dependent on lamin A. (A) Immunofluorescence analysis of SUN2 in embryonic fibroblasts derived from wild-type (+/+) and *Lmna* knockout (−/−) mice. SUN2 appeared as a rim-like shape around the nucleus in wild-type cells (left). While in *Lmna* ^−/−^ cells, aberrant distribution of SUN2 was detected in the cytoplasm (middle, arrow) and the irregularly shaped nucleus (right, arrowhead). (B) In the EGFP-Lamin A positive *Lmna* ^−/−^ embryonic fibroblast, SUN2 (red) restored its rim-like shape around the nucleus (arrow), whereas SUN2 localized predominantly in the cytoplasm of the lamin A negative cell (arrowhead). (C) SUN2 expression (green) remained aberrant in the *Lmna* ^−/−^ cell transfected with pDsRed-laminC (arrow). Nuclei were stained with DAPI. Scale bars, 20 µm.

Lamins are composed of A and B types [22]. A-type lamins, including lamin A and C, are alternative splicing products of *Lmna* at its 3′end [23]. Lamin A/C is required for the NE localization of SUN2 and we substantiate this by analyzing the distribution patterns of SUN2 in primary embryonic fibroblasts derived from wild-type and homozygous *Lmna* ^−/−^ mice [24]. In wild-type cells, SUN2 was localized mainly on the NE (Fig. 2A, left). In *Lmna* ^−/−^ cells, we observed three aberrant distribution patterns of SUN2, (1) streaks of SUN2 in the cytoplasm (Fig. 2A, arrow); (2) nuclear SUN2 was expressed but lost from one pole of the irregularly shaped nucleus (Fig. 2A, arrowhead); (3) same intensity of SUN2 staining in the nucleus and cytoplasm (not shown). The loss of expression from one pole of the nucleus in *Lmna* ^−/−^ cells has also been reported for lamin-associated protein 2, lamin B and Nup153 [24]. From three independent experiments, the percentage distribution of these three patterns was 56±7.6 %, 17±2.8 % and 14±3.1 % (100 cells were scored each time). In the remaining 13±2.6 % of the cells, the typical NE localization of SUN2 was observed, but the expression level was generally weaker than that observed in the wild-type. We further determined whether lamin A/C could reverse the aberrant distribution of SUN2 in *Lmna* ^−/−^ cells. In around 50 % of lamin A positive cells after transfection, SUN2 displayed the typical NE localization pattern (Fig. 2B, arrow), while the displacement of SUN2 was not reverted in the lamin A negative cell (Fig. 2B, arrowhead). In contrast, exogenous lamin C failed to restore the NE localization of SUN2 or itself in the lamin A negative cell (Fig. 2C, arrow).

### SUN2 is redistributed to endosomes upon overexpression of Rab5

**Figure 3:**
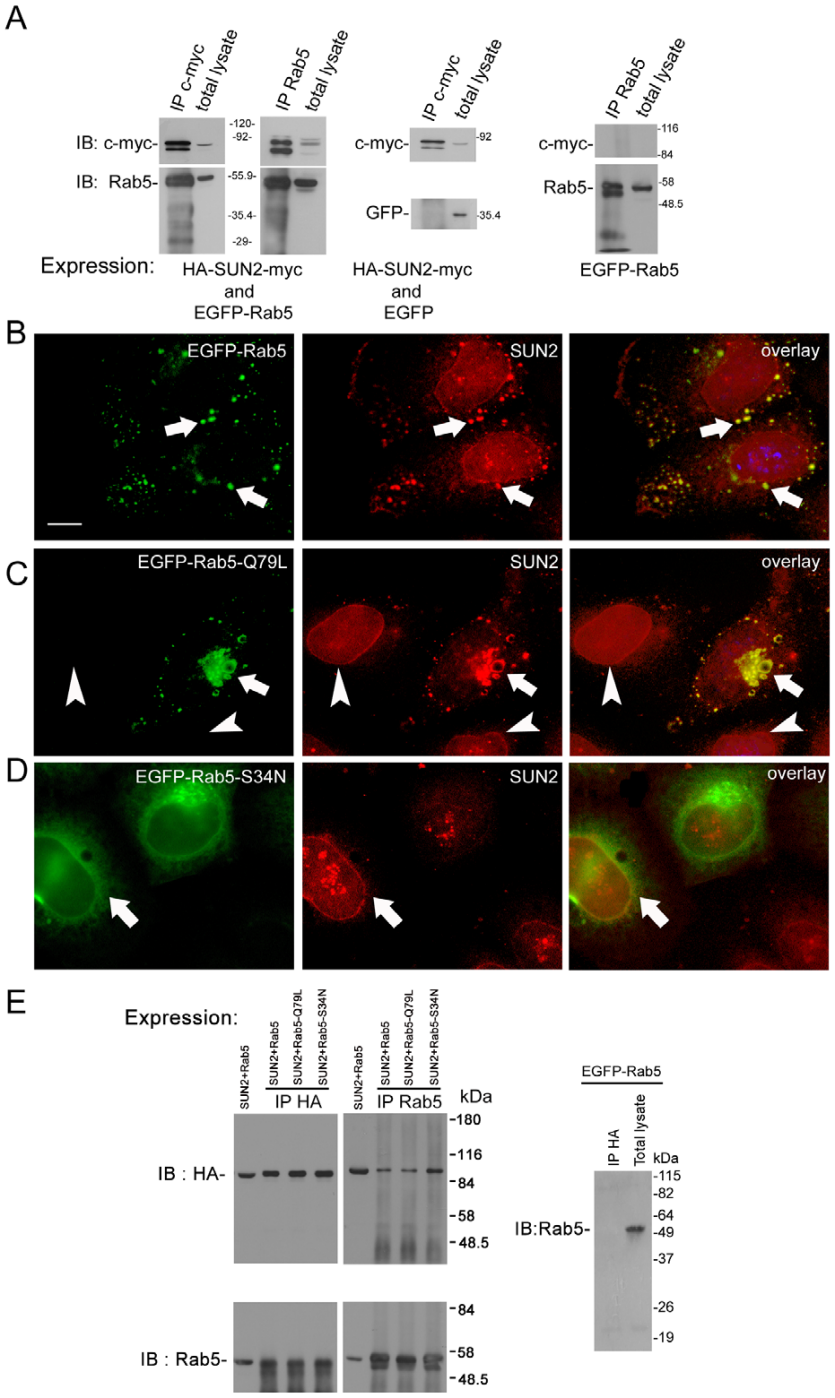
Rab5 and the GTPase-deficient mutant, Rab5^Q79L^, lead to SUN2 redistribution to endosomes. (A) Left: SUN2 coimmunoprecipitated with Rab5, using antibodies against c-myc and Rab5, in the protein lysate from HeLa cells cotransfected with pcDNA3-HA-SUN2-myc and pEGFP-Rab5. Middle: No binding between SUN2 and EGFP was observed in cells overexpressing HA-SUN2-myc and EGFP. Right: The antibody against c-myc could not recognize any bands in HeLa cells transfected with pEGFP-Rab5 only. Molecular weight is shown in kDa. (B) Immunofluorescence staining reveals that endogenous SUN2 partially colocalized with transfected EGFP-Rab5 in endosomes (arrows). (C) In the cell expressing pEGFP-Rab5^Q79L^, SUN2 was relocated to the enlarged endosomes from the NE (arrow), but not in Rab5^Q79L^-negative cells (arrowheads). (D) In Rab5^S34N^-positive cells, SUN2 remained on the NE (arrow). Scale bar, 10 µm. (E) Antibodies against HA and Rab5 were used as precipitating/immunoblotting antibodies in HeLa cells cotransfected with plasmids abbreviated on the top. HA-SUN2 coimmunoprecipitated with wild type or mutant forms of EGFP-Rab5. In the control shown on the right, the antibody against HA did not immunoprecipitate Rab5 in HeLa cells transfected with pEGFP-Rab5 only. IB, immunoblotting; IP, immunoprecipitation.

**Figure 4:**
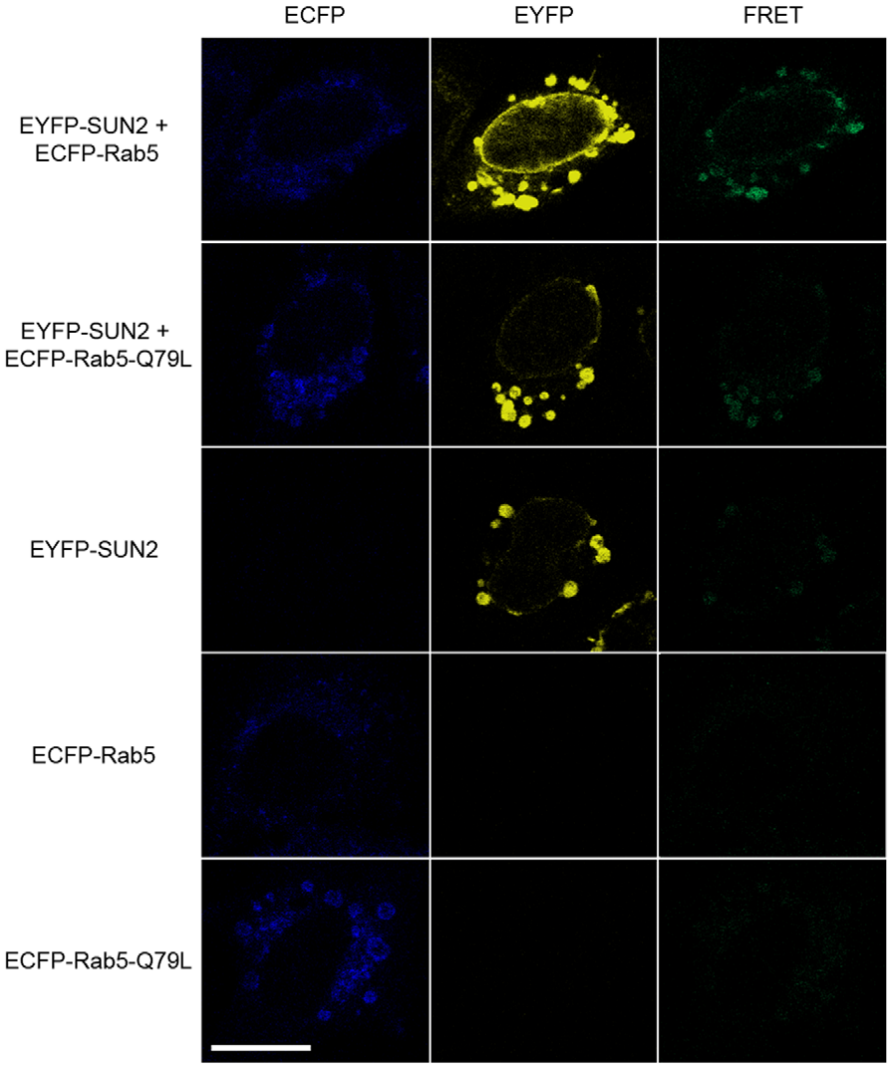
SUN2 and Rab5 interact in close proximity as detected by FRET assay. HeLa cells were transfected with plasmids encoding the protein indicated on the left. Shown are representative images acquired using the filter channels indicated on the top. FRET signal was detected in cells expressing both EYFP- SUN2 and ECFP-Rab5 (first row), or both EYFP-SUN2 and ECFP-Rab5^Q79L^ (second row). In singly transfected cells (third to fifth rows), no FRET signal could be detected when fluorescent images were acquired under identical conditions. Scale bar, 20 µm.

Unlike most other INM proteins exhibiting an exclusive NE localization, we observed punctate SUN2 staining in addition to the NE staining in a small proportion of HeLa cells, which resembled cytoplasmic vesicles. This staining pattern can also be found in other reports but is essentially ignored [11], [12], [25]. A previous study has shown that truncated SUN2 localizes in Rab5-positive vesicles in cotransfected HeLa cells. Furthermore, endogenous SUN2 is found in the membrane fraction after removal of nuclei [16]. By immunoprecipitation, we confirmed the interaction between full length SUN2 and Rab5 in HeLa cells overexpressing SUN2 and EGFP-Rab5 (Fig. 3A). Rab5 is a key regulator of early endocytosis. To further analyze the involvement of endogenous SUN2 in endocytosis, we used HeLa cells transiently transfected with EGFP-Rab5 and its two mutants. Rab5^Q79L^ is a GTPase-deficient mutant which stimulates endosome fusion, whereas Rab5^S34N^ is a dominant negative mutant preferentially GDP-bound [26]. Colocalization of endogenous SUN2 with Rab5 in endosomes was detected in cells overexpressing Rab5 (Fig. 3B). In cells expressing Rab5^Q79L^, SUN2 expression overlapped with Rab5^Q79L^ in enlarged early endosomes (Fig. 3C, arrow). In contrast, nontransfected HeLa cells maintained a uniform expression of SUN2 on the NE (Fig. 3C, arrowheads). SUN2 did not colocalize with Rab5^S34N^ but was found on the NE and some punctate structures in the nucleus (Fig. 3D, arrow). However, all the Rab5 mutant forms could interact with SUN2 (Fig. 3E). Close proximity between Rab5 and SUN2 (<10 Å) was confirmed by FRET. Using wildtype Rab5 and its mutant Rab5^Q79L^, the results confirm direct interaction between SUN2 and the GTP-bound form of Rab5 (Fig. 4).

**Figure 5:**
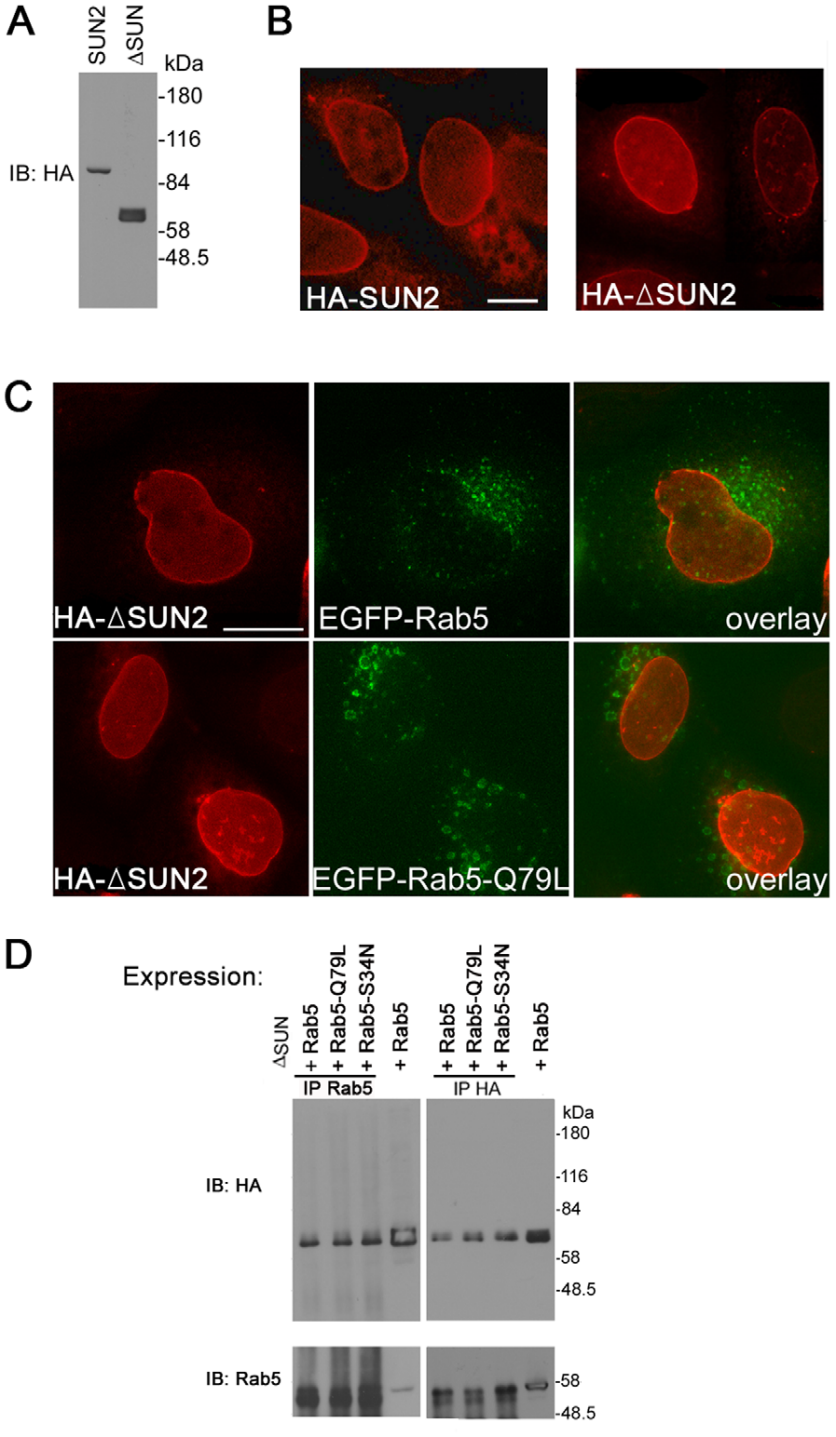
SUN domain is required for the translocation of SUN2 from the NE to the cytoplasm upon Rab5 overexpression. (A) HeLa cells transfected with pcDNA3-HA-SUN2-myc (SUN2) or pcDNA3-HA-ΔSUN (ΔSUN) were analyzed by the antibody against HA. ΔSUN is identified at ∼60 kDa; IB, immunoblotting. (B) Immunofluoresence staining using the antibody against HA shows that both full length HA-SUN2 and HA-ΔSUN localized on the NE of transfected HeLa cells. Scale bar, 10 µm. (C) HA-ΔSUN detected by anti-HA localized on the NE in HeLa cells transfected with either pEGFP-Rab5 or pEGFP-Rab5^Q79L^. Scale bar, 20 µm. (D) Antibodies against HA and Rab5 were used as precipitating (IP) / IB anibodies in cotransfected HeLa cells. HA-ΔSUN coimmunoprecipitated with EGFP-Rab5 and its mutant forms.

To investigate the importance of the SUN domain, a SUN2 construct with SUN domain deletion as shown in Figure 1A was generated. The anti-HA antibody efficiently identified this truncated protein, termed ΔSUN at its calculated size of ∼60 kDa (Fig. 5A). Like the full length protein, ΔSUN localized mainly to the NE, together with some cytoplasmic vesicles (Fig. 5B). In contrast to the endogenous SUN2, ΔSUN was concentrated at the NE after transfection with either Rab5 or its positive mutant (Fig. 5C). The result of coimmunoprecipitation showed ΔSUN interacted with Rab5 and its two mutants *in vitro* (Fig. 5D). This suggests that the SUN domain is not essential for the binding between SUN2 and Rab5, but it is required for the translocation of SUN2 to the cyotplasmic vesicles.

**Figure 6:**
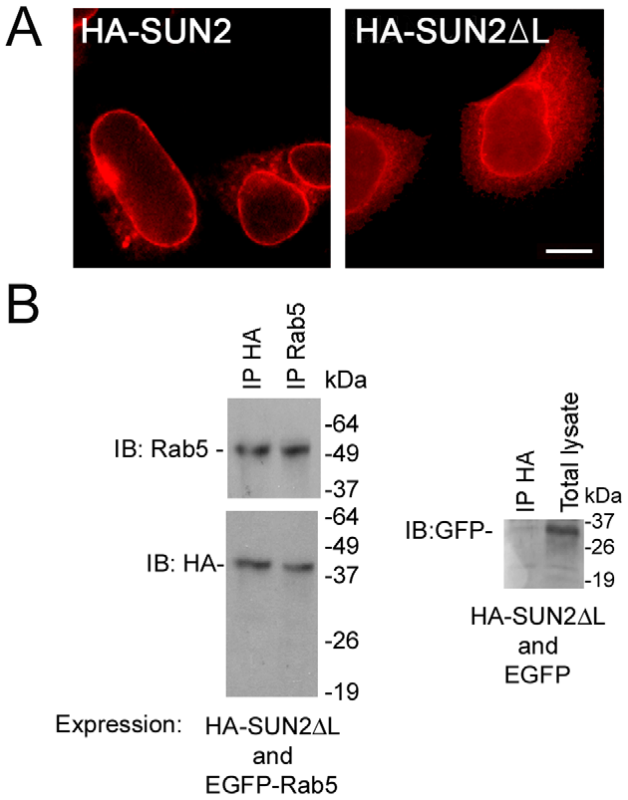
The N-terminus of SUN2 (SUN2ΔL) can interact with Rab5. (A) HA-SUN2ΔL (right) localized to both the NE and cytoplasm. Full length SUN2 is shown on the left. Scale bar, 10 µm. (B) HA-SUN2ΔL coimmunoprecipitated with EGFP-Rab5. Negative control is shown on the right.

A previous study has shown that the C-terminus of SUN2 can interact with Rab5 [16]. This raises the question of the physiologic significance of such an interaction between the luminal domain of SUN2 and Rab5, a cytoplasmic protein. To reconcile how Rab5 can affect the localization of endogenous SUN2, we looked into the interaction between the N-terminus of SUN2 (SUN2ΔL) and Rab5. SUN2ΔL localized to both the NE and cytoplasm (Fig. 6A). SUN2ΔL coimmunoprecipitated with Rab5-EGFP, but not EGFP, in cotransfected cells (Fig. 6B). This data suggest possible interaction between Rab5 and SUN2 under physiological conditions.

### SUN2 and endogenous Rab5 colocalize in endosomes

**Figure 7:**
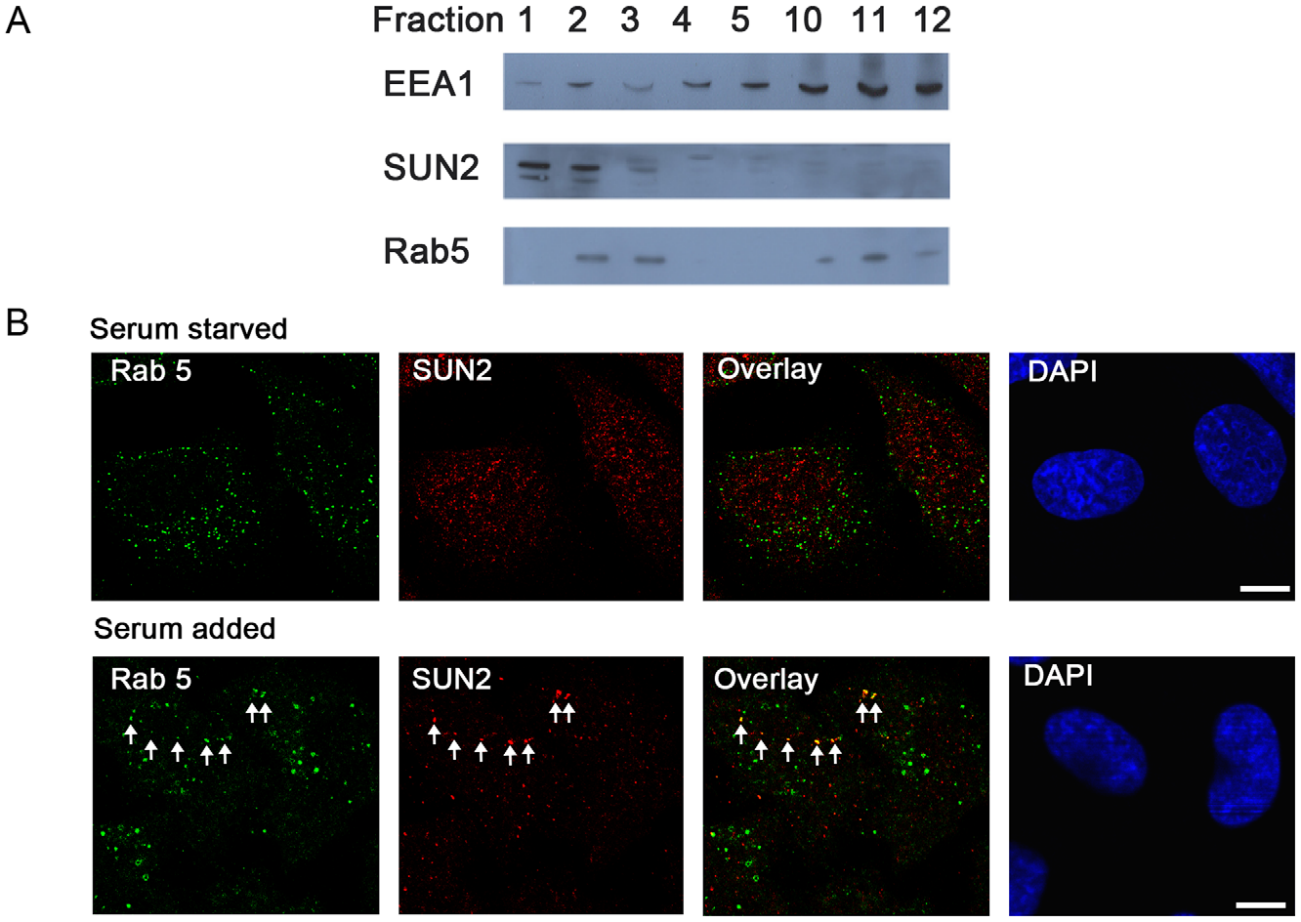
Endogenous SUN2 and Rab5 colocalize. (A) Western blot analysis of HeLa cell fractions separated by sucrose density gradient centrifugation with the antibodies indicated on the left. Twelve 1 ml fractions were collected from the top (fraction 1) to the bottom of the gradient. SUN2 and Rab5 coexisted in fractions 2 and 3. (B) HeLa cells were serum starved for 3 hours and then serum was added to induce endocytosis. Confocal images of double staining for SUN2 and Rab5 were taken before and 10 minutes after serum addition. Colocalization is indicated by arrows. Scale bar, 10 µm.

We moved on to investigate whether endogenous SUN2 and Rab5 colocalize. In subcellular fractionation experiment using sucrose float-up gradients, the early endosome marker EEA1 is concentrated in the dense fractions 10–12 whereas the top fractions 1–3 are highly enriched in Golgi apparatus, endosomes and associated vesicles. Localization of SUN2 and Rab5 overlapped in fractions 2 and 3 of nontransfected HeLa cells, indicating that partial colocalization of the two proteins in vesicles is possible (Fig. 7A). To initiate endocytosis, we serum starved the cells for 3 hours and then added 10% serum. Interestingly, the rim-like NE SUN2 staining decreased and prominent vesicle SUN2 staining appeared when cells were serum starved (Fig. 7B). Shortly after feeding with 10% serum, colocalization between endogenous SUN2 and Rab5 could be found in some vesicles (Fig. 7B, arrows).

### SUN2 affected transferrin-receptor mediated endocytosis

**Figure 8:**
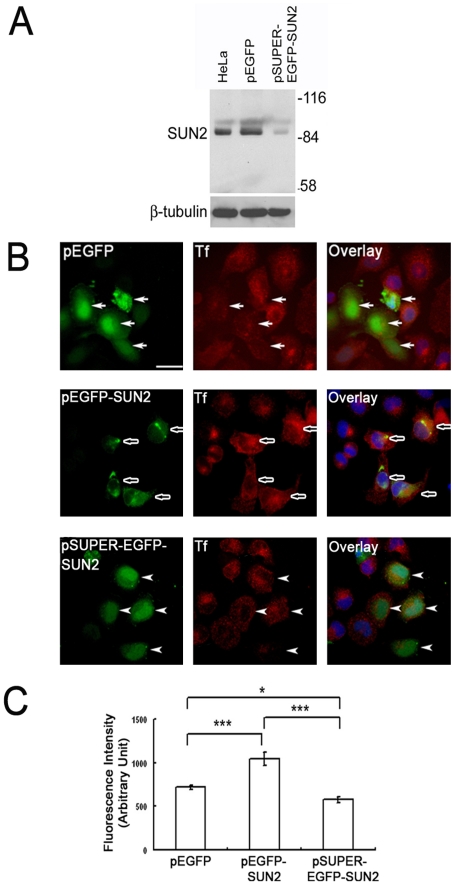
Knockdown of SUN2 expression attenuates transferrin uptake. (A) HeLa cells were transiently transfected with either pEGFP or pSUPER-EGFP-SUN2. SUN2 expression was significantly downregulated in cells transfected with pSUPER-EGFP-SUN2 as shown by Western blotting. (B) Texas Red labeled-Tf accumulation in HeLa cells transfected with the indicated plasmids. Fluorescent Tf in pEGFP transfected HeLa cells (arrows, top panel) was at a similar intensity as in nontransfected cells. Tf accumulation increased remarkably in cells transfected with pEGFP-SUN2 (open arrows, middle panel) and decreased in some but not all cells transfected with pSUPER-EGFP-SUN2 (arrowheads, bottom panel). Scale bar, 20 µm. (C) Statistical analysis was used to compare the effect of SUN2 on Tf uptake. Each group represents the mean ± S.E.M. of three independent experiments. *P<0.05, ***P<0.001, one way ANOVA with post Tukey test.

As Rab5 is a key molecule in endocytosis, we determined what role SUN2 plays in endocytosis. In a previous report, SUN2 has been shown to stimulate horseradish peroxidase accumulation when overexpressed. Conversely, addition of antiserum against SUN2 inhibited *in vitro* endosome fusion [16]. Here we transfected pSUPER-EGFP-SUN2 for expressing shRNA to knockdown SUN2 expression. Western blot analysis revealed that SUN2 expression was inhibited to a significant extent in transiently transfected HeLa cells (Fig. 8A). Next, transferrin uptake assay was employed. As shown in Fig. 8B, HeLa cells transfected with pEGFP-SUN2 showed a marked increase in Tf uptake, while most cells transfected with pSUPER-EGFP-SUN2 did not show an obvious decrease in the uptake of Tf compared to nontransfected cells. The immunofluorescence intensity of Tf inside each cell was further calculated by the confocal microscope software. Statistical analysis showed that overexpression of SUN2 significantly stimulated Tf internalization (P<0.001), while suppression of SUN2 expression attenuated the process (P<0.05) (Fig. 8C). Ideally, the experiment should be repeated in SUN2 knockout cells to see if a more significant effect than knockdown is revealed. The effects of forced and suppressed SUN2 expression on Tf uptake strongly suggest that SUN2 enhances receptor mediated endocytosis.

## Discussion

Both SUN1 and SUN2 are inner nuclear membrane proteins that serve to link the nuclear lamina and the cytoskeleton [9], [10], [12]. Besides, SUN domain proteins are found outside the nucleus. SUN4 is an outer dense fiber protein in sperm tail [6]. In S. pombe, sad1 is located on the spindle pole body, which is equivalent to the centrosome in mammalian cells [27]. SUN1 and SUN2 can also be detected in the centrosome [25], and SUN2 in vesicles as shown in the present study. We found that SUN2 is partly dependent on lamin A for NE retention and recruited to endosomes by the GTP-bound form of Rab5. Our approach has been emphasized on endogenous SUN2 because this is more likely to reflect its biologic function and extends the work using exogenous, truncated SUN2 [16].

To test whether the NE localization of SUN2 is dependent on an intact lamina, we examined in detail the localization of SUN2 in *Lmna* ^−/−^ cells. Around 13 % of these cells showed NE localization of SUN2. The fact that typical NE localization can be observed although only in a minority of *Lmna* ^−/−^ cells supports the notion that SUN2 is transported to the NE but not properly retained there in the absence of lamin A/C. Reintroduction of lamin A can restore the NE localization of SUN2 in 50 % of cells expressing lamin A, indicating that the stable retention of SUN2 on the NE is not just dependent on lamin A. By contrast, SUN1 localization is not affected in *Lmna* null or knockdown cells [12], [28]. As SUN1 interacts preferentially with prelamin A instead of mature lamin A, it may have a specific role in the processing of lamin A and in laminopathy [9], [12], [29]. Furthermore, SUN1 but not SUN2 has a specific role in determining the distribution of nuclear pore complex [5]. In contrast to SUN1, SUN2 does not require the predicted transmembrane region for its localization to the NE. This raises the possibility that SUN2 localizes to the NE through association with other NE-localized proteins [25], [30].

Here we show that SUN2 can partition between the NE and vesicle structures, which requires the SUN domain. Unlike endogenous SUN2, ΔSUN is not translocated to endosomes upon transfection with Rab5 or its GTPase-defective mutant Rab5^Q79L^ even though they coimmunoprecipitate (Fig. 5). This is reminiscent of a report on UNC-84, which shows that the SUN domain is required for *in vivo* function, but other domains are sufficient for *in vitro* interaction [8]. Previously it has been shown that truncated SUN2 colocalizes poorly with Rab5^Q79L^ [16] but we demonstrate that this is not the case for full length SUN2 and further show that the protein interaction is direct by FRET assay (Fig. 4). Until now most identified Rab5 effectors, including EEA1 and Rabenosyn-5, are recruited by the GTP-bound form of Rab5 [31]. Our data suggest that SUN2 also acts as a Rab5 effector via direct interaction with the GTP-bound form of Rab5.

The dual location of SUN2 in the cell is reminiscent of two other proteins, APPL (Adaptor protein containing PH domain, PTB domain and Leucine zipper motif) 1 and Alfy (Autophagy-linked FYVE protein). APPL1, identified as a Rab5 effector, localizes to endosomal compartments and translocates to the nucleus in response to the overexpression of a Rab5 GTPase activating protein or extracellular stimuli, such as epidermal growth factor and oxidative stress. APPL1 dissociation from endosomes depends on Rab5-GTP hydrolysis [32]. Alfy localizes mainly on the NE, and accumulates on the autophagic vesicles upon the induction of autophagocytosis [33]. Similarly, we found that SUN2 was redistributed dramatically to vesicles upon nutrient starvation which induces autophagy (data not shown). Upon serum starvation, we observed a shift of NE to cytoplamic EGFP-tagged SUN2 by live imaging (Fig. S1). In contrast to APPL1 and Alfy, SUN2 is a transmembrane protein. It is unlikely that transmembrane SUN2 travels from the NE to vesicles. We propose that a population of SUN2 is associated with the NE without being inserted into the membrane, as supported by the founding that SUN2 localization to the NE does not require the transmembrane domain [25], [30]. If this is the case, all three large proteins APPL1, Alfy and SUN2 are transported in and out of the nuclear membrane as membrane associated proteins upon external stimuli. A second possibility is vesicular SUN2 does not come from the NE. The exact mechanism of how SUN2 is distributed to vesicles in response to external stimuli warrants further investigation.

## Supporting Information

Figure S1 Shift of NE SUN2-EGFP upon serum starvation. HeLa cells were transfected with pEGFP-N1-SUN2. Two days after transfection, the medium was changed to DMEM only to start live cell microscopy on the motorized stage of a Zeiss Axiovert microscope. Images were acquired at 10 minute-intervals with Hamamatsu Orca camera (Hamamatsu Photonics). MetaMorph imaging software (Molecular Devices) was used for acquisition, assembly and deconvolution of acquired time lapse images. Time of serum starvation was recorded in minutes. Images produced by Dr. Cornelia Man, Department of Applied Biology & Chemical Technology, The Hong Kong Polytechnic University.
(TIF)
