# Supplementary figures and images for: Subcellular Localization of SUN2 Is Regulated by Lamin A and Rab5
Source: PLoS One. 2011 May 31;6(5):e20507. doi: 10.1371/journal.pone.0020507 (PMC3105078; doi:10.1371/journal.pone.0020507)

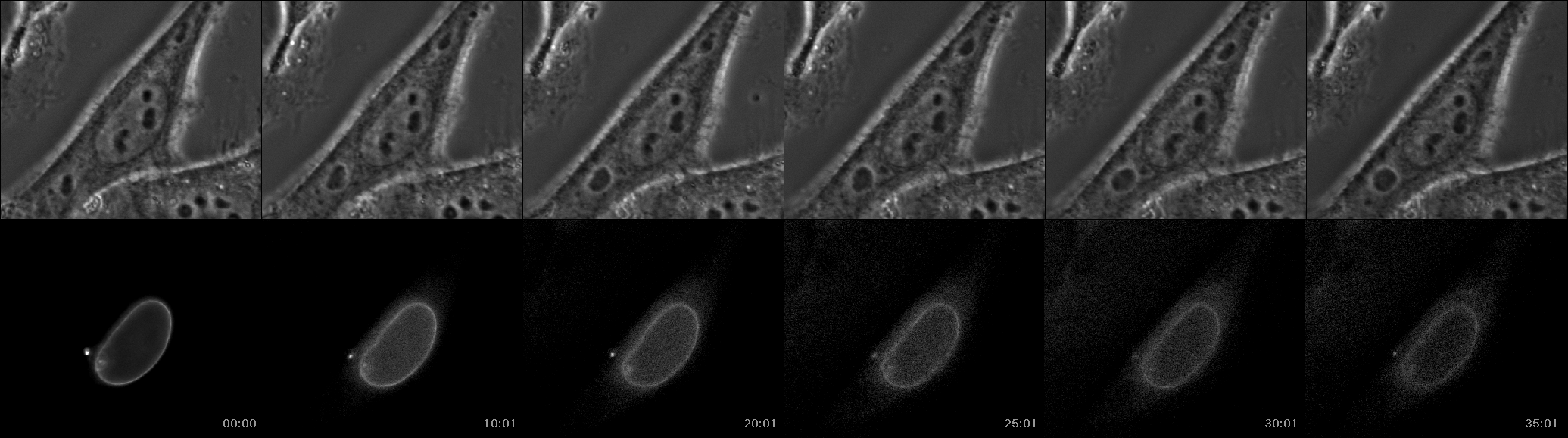

Supplement: Figure S1 — Shift of NE SUN2-EGFP upon serum starvation. HeLa cells were transfected with pEGFP-N1-SUN2. Two days after transfection, the medium was changed to DMEM only to start live cell microscopy on the motorized stage of a Zeiss Axiovert microscope. Images were acquired at 10 minute-intervals with Hamamatsu Orca camera (Hamamatsu Photonics). MetaMorph imaging software (Molecular Devices) was used for acquisition, assembly and deconvolution of acquired time lapse images. Time of serum starvation was recorded in minutes. Images produced by Dr. Cornelia Man, Department of Applied Biology & Chemical Technology, The Hong Kong Polytechnic University. (TIF) [file pone.0020507.s001.tif]
